# Reduction in Adiposity, β-Cell Function, Insulin Sensitivity, and Cardiovascular Risk Factors: A Prospective Study among Japanese with Obesity

**DOI:** 10.1371/journal.pone.0057964

**Published:** 2013-03-06

**Authors:** Maki Goto, Akemi Morita, Atsushi Goto, Kijo Deura, Satoshi Sasaki, Naomi Aiba, Takuro Shimbo, Yasuo Terauchi, Motohiko Miyachi, Mitsuhiko Noda, Shaw Watanabe, for the SCOP Study Group

**Affiliations:** 1 Department of Diabetes Research, Diabetes Research Center, National Center for Global Health and Medicine, Tokyo, Japan; 2 Department of Endocrinology and Metabolism, Yokohama City University Graduate School of Medicine, Yokohama, Japan; 3 National Institute of Health and Nutrition, Tokyo, Japan; 4 Department of Nutrition, College of Nutrition, Koshien University, Hyogo, Japan; 5 Saku Central Hospital, Nagano, Japan; 6 Department of Social and Preventive Epidemiology, School of Public Health, University of Tokyo, Tokyo, Japan; 7 Department of Nutrition and Life Science, Kanagawa Institute of Technology, Kanagawa, Japan; 8 Department of Clinical Research and Informatics, National Center for Global Health and Medicine, Tokyo, Japan; 9 Department of Diabetes and Metabolic Medicine, Center Hospital, National Center for Global Health and Medicine, Tokyo, Japan; John Hopkins University School of Medicine, United States of america

## Abstract

**Background:**

A reduction in adiposity may be associated with an improvement in insulin sensitivity and β-cell function as well as cardiovascular disease (CVD) risk factors; however, few studies have investigated these associations in a longitudinal setting.

**Methods:**

To investigate these associations over a 1-year period, we conducted an observational analysis of 196 Japanese subjects with obesity in the Saku Control Obesity Program. We investigated the relations between changes in adiposity (body mass index [BMI], waist circumference, subcutaneous fat area [SFAT], and visceral fat area [VFAT]) and changes in HbA1c, fasting plasma glucose (FPG), insulin sensitivity index (ISI), the homeostasis model assessment β cell function (HOMA-β), lipids, and blood pressure.

**Results:**

All adiposity changes were positively associated with HbA1c and FPG changes. Reductions in BMI and VFAT were associated with HOMA-β reduction. Reductions in all adiposity measures were associated with an improvement in the ISI. Changes in most adiposity measures were positively associated with changes in blood pressure and lipid levels, except for LDL.

**Conclusion:**

The present findings provide additional supportive evidence indicating that a reduction in adiposity may lead to an improvement in insulin sensitivity and the reduction of CVD risk factors in obese individuals.

## Introduction

Since the identification of obesity as the a strong risk factor for various diseases, such as cardiovascular disease (CVD), stroke, and type 2 diabetes, lifestyle modification and weight loss have become major strategies for disease prevention. The development of CVD may be mediated through obesity, decreased insulin sensitivity, dyslipidemia, or high blood pressure [1]-[3]. Previous studies have investigated the associations between obesity and CVD risk factors [2], [4]-[7], but few studies have examined the associations between changes in adiposity measures and changes in CVD risk factors [8], [9]. Furthermore, simple adiposity assessments (e.g., body mass index [BMI], waist circumference, or waist-to-hip ratio) might underestimate the contribution of adiposity to the risk of CVD [10]. Therefore, the use of the visceral fat area (VFAT) and the subcutaneous fat area (SFAT) determined using computed tomography might detect important relations between changes in adiposity and changes in CVD risk factors [9].

In light of effective diabetes prevention, strategies for preserving β-cell function are of great importance. It has now been recognized that a compensatory period (i.e., a compensatory increase in insulin secretion secondary to insulin resistance with little change in the glucose levels) exists before the diagnosis of diabetes, and the insulin secretion decreases after the diagnosis of diabetes [11]. Insulin resistance is considered to be responsible for the increase in insulin levels during the compensatory period. Therefore, it is of great interest to investigate whether reduction in adiposity is associated with improvement in insulin secretion as well as insulin resistance. However, the relationship between the changes in adiposity and insulin resistance or β-cell function has not been well-understood.

Therefore, we investigated the associations between changes in adiposity, including SFAT and VFAT evaluated using computed tomography, and changes in insulin secretion and sensitivity as well as CVD risk factors in 196 Japanese participants with obesity over a 1-year period.

## Materials and Methods

### Ethics statement

This study was reviewed and approved by the Ethical Committee of the National Institute of Health and Nutrition and Saku Central Hospital. Participants received a precise explanation of the study and provided their written informed consent.

### Subjects

This study is a secondary analysis of a randomized controlled trial, the Saku Control Obesity Program (SCOP), examining the effect of behavioral treatment and exercise at the Saku Central Hospital Human Dock Center. The details and design of the study have been previously described elsewhere [12 reatment at the Saku Health Dock Center. People who had undergone health checkups at the center were registered in the database, and 976 members between the ages of 40 and 64 years who did not have type 1 diabetes or severe diseases, such as stroke, cardiovascular disease, advanced cancer or significant renal or hepatic dysfunction, and had a body mass index (BMI) in the upper five percentile of all examinees (28.3 or above) were invited. A total of 237 people participated in the study and were randomly assigned to two groups: group A, who participated in a lifestyle intervention program during year 1; and group B, who participated in the same intervention program during year 2. The intervention group received individual-based counseling on diet and physical activity, and by 1 year, the intervention group had significantly lost more weight than the control group (−5.0 kg vs. 0.1 kg among men, P < 0.01; −2.9 kg vs. −0.2 kg among women, P < 0.01) [15].

In this study, we excluded 29 patients who had already been diagnosed as having diabetes and were receiving treatment at baseline or at 1 year (27 participants and 2 participants, respectively), 3 participants not in fasting states at the time of blood sampling, and 9 participants who did not complete the study. Finally, 196 participants were included in the analysis.

### Anthropometric Measurements

The height (cm) and weight (kg) of the subjects were measured using an automatic scale (BF-220; Tanita, Tokyo, Japan), and the BMI was calculated as the weight (kg) divided by the squared height (m^2^). The waist circumference was measured twice in a standing position at the umbilicus level using a fiber glass measuring tape, and the average was used for the analysis. Blood pressure was measured in a sitting position using a validated automated blood pressure monitor (HEM-907; Omron, Kyoto, Japan) [16].

The VFAT and SFAT were assessed based on a CT scan at the level of the umbilicus while the subject was in a supine position (Fat scan; N2 system Corp., Japan) [6]. Physical activity levels were obtained from a questionnaire that aimed to identify activity levels during the recent month as follows: light activity, approximately 1 hour of walking or 3 hours of standing per day; medium activity, approximately 2 hours of walking or 6 to 7 hours of standing per day; moderately-to-heavy activity, approximately 1 hour of intense activity plus 9 hours of walking or standing per day; heavy activity, approximately 2 hours of intense activity plus 9 hours of walking or standing per day.

### Laboratory Procedures

Following an overnight fast, blood samples were collected at the time of each health checkup at the Saku Health Dock Center. Blood samples were collected in tubes containing EDTA and heparin for the measurement of fasting plasma glucose (FPG), insulin, and HbA1c and in serum gel separator tubes for the measurement of total cholesterol, high-density lipoprotein cholesterol (HDL), and triglyceride (TG) levels. Routine laboratory blood analyses were performed at the Saku Central Hospital. HbA1c levels were measured using a high-performance liquid chromatography method (TOSOH HLC-723 G8, Tosoh Corporation, Tokyo, Japan) with intra- and inter-assay coefficients of variation (CVs) ≤1.4%. The plasma glucose levels were analyzed using an enzymatic method (ECO glucose buffer; A&T Corporation, Kanagawa, Japan), with intra- and inter-assay CVs ≤0.8%. The plasma insulin levels were analyzed using an electrochemiluminescence immunoassay (Modular E170; Roche Diagnostics, Mannheim, Germany) with intra- and inter-assay CVs ≤3.6%. The serum total cholesterol, HDL, and TG concentrations were determined using enzymatic methods (Detaminar L TC II, Kyowa Medex, Tokyo, Japan; Cholestest N HDL, Sekisui Medical Co Ltd, Tokyo, Japan; and Mizuho TG-FR Type II, Mizuho Medi, Saga, Japan, respectively) and an autoanalyzer BM-2250 (Nihon Denshi, Tokyo, Japan) with intra- and inter-assay CVs of ≤2.3%. The HbA1c values were determined as Japan Diabetes Society (JDS) values and then converted to National Glycohemoglobin Standardization Program (NGSP) values using the following conversion formula: HbA1c (NGSP)  =  1.02×HbA1c (JDS)+0.25% [17]. The low-density lipoprotein cholesterol levels (LDL) were calculated using the Friedewald equation: LDL  =  total cholesterol-(HDL + [TG/5]).

To evaluate insulin sensitivity, we used the homeostasis model assessment for insulin resistance (HOMA-IR)[18] and the insulin sensitivity index (ISI) with reduced time points [19]; to evaluate β cell function, we used the homeostasis model assessment β cell function (HOMA-β)[20] and the insulinogenic index[21], [22] based on a 75 g oral glucose tolerance test (OGTT). The HOMA-IR was calculated as follows: fasting insulin (I_0_) (µIU/ml) × fasting glucose (G_0_) (mg/dL)/405 [18]. We calculated the ISI using reduced time points because of the absence of 90-minute glucose-load values from the OGTTs, with the measurement of the insulin and glucose levels at 0, 60 and 120 minutes as follows: ISI  =  10,000/[square root of (G_0_ × I_0_ × mean OGTT glucose concentration × mean OGTT insulin concentration)] [23]. This index has been reported to be well correlated with the original composite index; we did not use G_30_ and I_30_ in our calculations because this time point was not recommended when using reduced time points to calculate the ISI [23]. The HOMA-β was calculated using the formula: 360 × I_0_ (µIU/mL)/[G_0_ (mg/dL) −63]. The insulinogenic index was calculated as follows: (I_30_–I_0_)/(G_30_–G_0_) [24].

### Data analysis

The characteristics of the study population are presented as the mean for continuous variables. We calculated the means (± SDs) and the proportions of the covariates. A visual inspection of the histograms did not reveal any violations of the assumption of normality; we conducted the analysis without the use of log transformation. To compare the characteristics of the participants at baseline and at 1 year, we used a paired *t* test (Table 1). Then, we investigated the associations of CVD risk factors and insulin secretion and sensitivity using adiposity measures (Tables 2, 3, 4). We used multiple linear regression models to estimate the point estimates, the 95% confidence intervals (95% CIs), and the *P*-values with adjustments for potential confounding factors including age, sex, baseline adiposity measures, baseline insulin related indexes, intervention group assignment, and physical activity level. In addition, we conducted the following sensitivity analyses: (i) stratification according to sex, (ii) stratification according to WHO diagnostic criteria for impaired glucose tolerance (IGT) and diabetes, and (iii) exclusion of participants with medications for hyperlipidemia. We also examined the associations with stratification according to the intervention assignment, but the direction and strength of the associations remained consistent across the subgroups. Thus, we focused primarily on the associations for the full sample (i.e., the product terms were not included in the models).

**Table 1:** Baseline characteristics of participants.

	Baseline	1 year	P value*
N	196	196	
Male/Female (n)	96/100		
Age (years)	53.8 ± 6.4		
BMI (kg/m^2^)	30.5 ± 3.1	29.6 ± 3.5	< 0.001
Subcutaneous fat area (cm^2^)	294 ± 102	268 ± 94	< 0.001
Visceral fat area (cm^2^)	139 ± 48	123 ± 47	< 0.001
Waist circumference (cm)	102 ± 8	100 ± 9	< 0.001
HbA1c (%)	6.0 ± 0.8	5.8 ± 0.5	< 0.001
Fasting plasma glucose (mg/dL)	106 ± 13	107 ± 14	0.21
Fasting insulin (µIU/mL)	11.3 ± 8.0	9.4 ± 10.0	0.01
HOMA-IR	3.0 ± 2.2	2.6 ± 3.3	0.14
HOMA-β	100 ± 72	77 ± 62	< 0.001
Insulin Sensitivity Index	4.8 ± 3.4	6.5 ± 4.2	< 0.001
Insulinogenic index	0.84 ± 0.90	0.91 ± 1.8	0.57
LDL (mg/dL)	126 ± 34	128 ± 30	0.37
HDL (mg/dL)	53 ± 12	52 ± 13	0.21
Triglyceride (mg/dL)	162 ± 106	142 ± 72	< 0.001
Systolic blood pressure (mmHg)	138 ± 19	132 ± 20	< 0.001
Diastolic blood pressure (mmHg)	85 ± 14	83 ± 14	0.07

Data are (*n*) or means ± SD. * *P* value for paired *t* test.

Abbreviations: BMI, body mass index; HOMA-IR, homeostasis model assessment ratio; HOMA-β, homeostasis model assessment β cell function.

**Table 2:** Associations between changes in adiposity measures and glucose indices from baseline to 1 year.

Outcome variables	Δ HbA1c (%)		Δ FPG (mg/dL)	
	Coef.	*P* value	Coef.	*P* value
Explanatory variables	(95% CL)		(95% CL)	
Δ BMI (kg/m^2^)	0.09	<0.001	1.90	0.001
	(0.05, 0.12)		(0.79, 3.02)	
Δ Subcutaneous fat area (cm^2^)	0.003	<0.001	0.07	0.003
	(0.002, 0.005)		(0.03, 0.12)	
Δ Visceral fat area (cm^2^)	0.005	<0.001	0.08	0.01
	(0.003, 0.007)		(0.02, 0.14)	
Δ Waist circumference (cm)	0.03	<0.001	0.48	0.01
	(0.01, 0.04)		(0.11, 0.86)	

Abbreviations: BMI, body mass index; FPG, fasting plasma glucose; Coef., coefficient; CL, confidence limit.

A multiple linear regression model was used to adjust for potential confounding factors including age, sex, physical activity levels, intervention assignment, the baseline adiposity measure of interest, and the baseline value of the outcome variable.

**Table 3:** Associations between changes in adiposity measures and insulin related indexes from baseline to 1 year.

Outcome variables	Δ Insulin (µIU/mL)		Δ HOMA-IR		Δ ISI		Δ HOMA-β		Δ Insulinogenic index	
	Coef.	*P* value	Coef.	*P* value	Coef.	*P* value	Coef.	*P* value	Coef.	*P* value
Explanatory variables	(95% CL)		(95% CL)		(95% CL)		(95% CL)		(95% CL)	
Δ BMI (kg/m^2^)	1.09	0.02	0.32	0.045	-1.05	<0.001	7.43	0.007	0.14	0.12
	(0.16, 2.01)		(0.01, 0.64)		(-1.32, -0.78)		(2.02, 12.8)		(-0.04, 0.31)	
Δ Subcutaneous fat area (cm^2^)	0.02	0.29	0.01	0.39	-0.03	<0.001	0.12	0.31	0.003	0.39
	(-0.02, 0.06)		(-0.01, 0.02)		(-0.04, -0.02)		(-0.11, 0.36)		(-0.004, 0.01)	
Δ Visceral fat area (cm^2^)	0.06	0.01	0.02	0.048	-0.05	<0.001	0.50	0.001	-0.0005	0.92
	(0.01, 0.11)		(0.0001, 0.03)		(-0.06, -0.03)		(0.21, 0.79)		(-0.01, 0.009)	
Δ Waist circumference (cm)	0.25	0.10	0.07	0.18	-0.29	<0.001	1.80	0.053	0.04	0.15
	(-0.05, 0.56)		(-0.03, 0.18)		(-0.39, -0.20)		(-0.02, 3.62)		(-0.02, 0.10)	

Abbreviations: BMI, body mass index; HOMA-IR, homeostatic model assessment for insulin resistance; ISI, insulin sensitivity index; HOMA-β, homeostasis model assessment for β-cell function; Coef., coefficient; CL, confidence limit.

A multiple linear regression model was used to adjust for potential confounding factors including age, sex, physical activity levels, intervention assignment, the baseline adiposity measure of interest, and the baseline value of insulin related indices.

**Table 4:** Associations between changes in adiposity measures and lipid levels and blood pressure from baseline to 1 year.

Outcome variables	Δ LDL		Δ HDL		Δ Triglyceride		Δ Systolic blood pressure		Δ Diastolic blood pressure	
	(mg/dL)		(mg/dL)		(mg/dL)		(mmHg)		(mmHg)	
	Coef.	*P* value	Coef.	*P* value	Coef.	*P* value	Coef.	*P* value	Coef.	*P* value
Explanatory variables	(95% CL)		(95% CL)		(95% CL)		(95% CL)		(95% CL)	
Δ BMI (kg/m^2^)	0.47	0.67	−0.63	0.06	9.37	<0.001	2.03	0.01	1.48	0.01
	(−1.71, 2.66)		(−1.28, 0.03)		(4.75, 14.0)		(0.47, 3.59)		(0.35, 2.62)	
Δ Subcutaneous	0.04	0.39	−0.01	0.49	0.19	0.06	0.03	0.32	0.03	0.19
fat area (cm^2^)	(−0.05, 0.13)		(−0.04, 0.02)		(−0.01, 0.39)		(−0.03, 0.10)		(−0.02, 0.08)	
Δ Visceral	−0.03	0.56	−0.05	0.01	0.48	<0.001	0.12	0.005	0.07	0.04
fat area (cm^2^)	(−0.15, 0.08)		(−0.08, −0.01)		(0.23, 0.72)		(0.04, 0.20)		(0.004, 0.13)	
Δ Waist	0.33	0.36	−0.17	0.11	2.61	0.001	0.66	0.01	0.45	0.02
circumference (cm)	(−0.38, 1.05)		(−0.39, 0.04)		(1.07, 4.15)		(0.15, 1.18)		(0.08, 0.82)	

Abbreviations: BMI, body mass index; LDL, low density cholesterol; HDL, high density cholesterol; Coef., coefficient; CL, confidence limit.

A multiple linear regression model was used to adjust for potential confounding factors including age, sex, physical activity levels, intervention assignment, the baseline adiposity measure of interest, and the baseline value of lipid levels.

Two-sided *P* values <0.05 were considered to be statistically significant. Analyses were performed using Stata software (version 12; Stata Corp, College Station, TX).

## Results

Among a total of 196 participants aged 40 to 64 years, the mean age was 53.8 years. At baseline, all the participants had obesity with relatively high average glucose levels accompanied by insulin resistance and hyperinsulinemia (Table 1). Over a 1-year period, reductions in all adiposity measures, HbA1c, insulin secretion, TG, and systolic blood pressure were observed, possibly because of the trial intervention (Table 1).

After adjustments for age, sex, physical activity, intervention assignment, baseline adiposity measures, and the baseline value of the outcome variable, reductions in all adiposity measures, including BMI, SFAT, VFAT, and waist circumference, were associated with reductions in the HbA1c and FPG levels (Table 2). The relations between the changes in adiposity and glucose levels were consistent with previous findings. In this study, we were able to further examine the associations of changes in adiposity with the changes in insulin secretion and resistance. As expected, reductions in BMI and VFAT were associated with an improvement in insulin sensitivity as measured using the HOMA-IR and ISI (Table 3). Furthermore, reductions in all adiposity measures were associated with an elevation in the ISI. Decreases in BMI and visceral fat area were related to reduced insulin secretion as measured using the HOMA-β. In contrast, changes in the insulinogenic index were not related to changes in any adiposity measure (Table 3).

Changes in any of the adiposity measures were not associated with the change in the LDL level, but a change in VFAT was inversely associated with a change in HDL while changes in the BMI, VFAT, and waist circumference were positively associated with a change in the TG level (Table 4). In addition, changes in the BMI, VFAT, and waist circumference were positively associated with changes in the systolic and diastolic blood pressure (Table 4).

A sensitivity analysis stratified according to sex did not substantially change the results; and furthermore, the exclusion of the participants with hyperlipidemia medications (n = 38) did not materially alter the results (data not shown). According to the WHO diagnostic criteria [25], there were 86 participants with normal glucose tolerance (NGT), 11 participants with impaired fasting glycemia (IFG), 76 participants with impaired glucose tolerance (IGT), and 23 participants with diabetes. The adiposity-HbA1c association was slightly stronger among the participants with IFG, IGT, and diabetes (*n*  =  110), compared with those with NGT. After excluding participants with diabetes, the associations between the changes in HOMA-β and the changes in adiposity measures (e.g., VFAT) became stronger (coefficient, 0.62; 95%CI 0.31, 0.92).

## Discussion

In this prospective study, changes in all the examined adiposity measures were positively associated with changes in glycemia in 196 Japanese participants with obesity. Furthermore, we found that reductions in BMI, visceral fat area, and waist circumference were associated with a reduction in HOMA-β, while no association was observed for the insulinogenic index. Reductions in BMI and VFAT were also associated with an improvement in insulin sensitivity. In addition, changes in adiposity were related to changes in TG and blood pressure. These findings suggest that controlling adiposity among obese individuals may effectively improve CVD risk factors and insulin sensitivity.

These results were consistent with a preceding study among obese individuals with type 2 diabetes, in which weight loss was associated with an improvement in glycemia, blood pressure, TG, and HDL [8], [9]. In the current study, we were able to assess VFAT and SFAT changes using computed tomography. We found that a reduction in visceral adiposity as well as the BMI was associated with an improvement in the CVD risk factors. Consistent with the previous study [8], the adiposity change was not associated with the LDL change in the current study. In another study reviewing weight loss effects and lipid outcomes [26], weight loss was reported to be associated with the LDL reduction. This dissociation may be because, in a previous review [26], studies with a large weight reduction (22 to 55 kg) as a result of surgical intervention were included. In the current study, the mean weight reduction within 1 year was 2.4 kg (SD 4.3), so the amount of the weight reduction may have been insufficient to detect any changes.

Among adiposity measures, changes in BMI and VFAT were positively associated with changes in insulin and HOMA-IR and were inversely associated with changes in ISI. Indeed, the change in ISI was inversely associated with changes in all the adiposity measures. These findings indicate that a reduction in adiposity may lead to an improvement in insulin sensitivity. This finding is consistent with a preceding study showing that baseline adiposity measures were inversely associated with future insulin sensitivity indices [6]. Although changes in SFAT and the waist circumference were also related to some of the insulin related indices, our findings may suggest that BMI and VFAT may be better markers of adiposity in this obese population. This finding is consistent with the results of preceding studies among Japanese-Americans, which have shown that the baseline VFAT, but not the SFAT, was associated with future insulin resistance [6].

Reductions in BMI, VFAT, and waist circumference were associated with a decrease in HOMA-β. Importantly, a previous study has shown that HOMA β-cell function, another measure of β-cell function, is elevated 3–4 years before the diagnosis of diabetes, and then decreases until the time of diagnosis, indicating that a period of compensation for insulin resistance exists [27]. In our study, a reduction in adiposity was related to a reduction in insulin resistance, which was accompanied by a reduction in HOMA-β. Furthermore, the associations between the HOMA-β change and the adiposity changes became stronger after the exclusion of participants with diabetes (coefficient for ΔVFAT-ΔHOMA-β, 0.50 to 0.62). This finding may suggest that a compensatory period actually exists among obese individuals who did not develop diabetes―and the adiposity reduction may reduce the needs for compensatory hyperinsulinemia.

Preceding studies have also reported that a decrease in the early-phase insulin response to glucose is a major feature of glucose tolerance among Japanese [22], [28], [29]. However, the change in the insulinogenic index was not related to changes in any adiposity measures in our study. This finding may suggest that the insulinogenic index may not measure compensatory hyperinsulinemia observed during the compensatory period, but might instead evaluate residual β-cell function. Thus, the null association between the insulinogenic index change and the adiposity change suggests that a reduction in adiposity may be insufficient to improve residual β-cell function.

Some limitations of the present study need to be addressed. First, participants in this study were Japanese individuals with obesity, and the sample size was limited; thus generalizability may be a problem. Second, although smoking has been reported to be related to CVD and weight reduction [2], smoking data was not available, and the analysis could not be adjusted for smoking. Third, we did not have intermediate measurements. Finally, because the follow up period was relatively short, a longer period observation period is needed to confirm the results. The major strengths of this study were the high compliance of our study participants, which enabled us to assess CVD risk factors, and the observation of adiposity changes using computed tomography over a 1-year period with few participant dropouts.

## Conclusions

Our findings suggest that the reduction of obesity, especially the BMI and visceral fat area, may be associated with an improvement in CVD risk factors. Furthermore, we found that reductions in BMI and visceral adiposity were associated with a reduced HOMA-β and HOMA-IR. These findings support the notion that controlling adiposity among obese individuals may effectively improve CVD risk factors and insulin resistance.
